# *Clcn3* deficiency ameliorates high-fat diet-induced obesity and improves metabolism in mice

**DOI:** 10.3389/fnut.2024.1387806

**Published:** 2024-05-09

**Authors:** Sirui Duan, Bo Li, Shiyu Cui, Yaoyao Chen, Ying He, Lihong Fan

**Affiliations:** ^1^Department of Cardiovascular Medicine, The First Affiliated Hospital of Xi’an Jiaotong University, Xi’an, China; ^2^Department of Cardiology, Ninth Hospital of Xi’an, Xi’an, China; ^3^Department of Pathology, School of Basic Medical Sciences, Xi’an Jiaotong University Health Science Center, Xi’an Jiaotong University, Xi’an, China; ^4^Graduate Students Teaching Experiment Center, Xi’an Jiaotong University Health Science Center, Xi’an, China

**Keywords:** *Clcn3*, high-fat diet, obesity, glucolipid metabolism, RNA seq, UCP1

## Abstract

**Objective:**

Obesity is defined as excess body fat and is a current health epidemic associated with increased risk for type 2 diabetes and cardiovascular disease. The ClC-3 chloride channel/antiporter, encoded by the *Clcn3*, is associated with some diseases, like carcinoma, nervous system diseases, and metabolic diseases. To verify the relationship between the *Clcn3* and weight including metabolic changes, searching for a new target for metabolic therapy of obesity, we designed the experiment.

**Methods:**

The mice were divided into 4 different groups: *Clcn3*^+/+^ mice + high-fat diet (HFD), *Clcn3*^−/−^ mice + HFD, *Clcn3*^+/+^ mice + normal diet (ND), *Clcn3*^−/−^ mice + ND, and fed for 16 weeks. After the glucose tolerance test and insulin tolerance test, peripheral blood and adipose tissues were collected. Moreover, we performed transcriptome sequencing for the epididymal white adipose tissue from *Clcn3*^+/+^ and *Clcn3*^−/−^ mice with the high-fat diet. Western blotting verified the changes in protein levels of relevant metabolic genes.

**Results:**

We found that the *Clcn3*^−/−^ mice had lower body weight and visceral fat, refining glucose and lipid metabolism in HFD-induced mice, but had no effect in normal diet mice. RNA-seq and Western blotting indicated that *Clcn3* deficiency may inhibit obesity through the AMPK-UCP1 axis.

**Conclusion:**

Modulation of *Clcn3* may provide an appealing therapeutic target for obesity and associated metabolic syndrome.

## 1 Introduction

Metabolic diseases have become a growing part of the global disease burden. A significant rise in metabolic risk represents a major health challenge. Obesity was the largest proportion of mortality related to metabolic diseases (40.36 and 41.83% of deaths in males and females, respectively) (1). With obesity being a common risk factor, these diseases often occur together, such as type 2 diabetes mellitus (T2DM), hypertension (HTN), hyperlipidemia (HLD), cardiovascular disease (CVD), and more. These exacerbate the disease burden of the population contributing to a decline in both quality of life and life expectancy (2).

Chloride ions are the most abundant anion and are involved in many different physiological and pathological processes. The molecular constituents of the volume-regulated anion channel (VRAC) are diverse, including LRRC8A-E, CFTR, ClCs, and more (3). The ClC-3 chloride channel, encoded by *Clcn3*, is critical for several basic cellular functions, such as cell volume regulation, proliferation, apoptosis, differentiation, and β cell insulin secretion (4). ClC-3 is expressed in most tissues, including the brain, retina, adrenal gland, pancreas, intestines, epididymis, kidney, liver, skeletal muscle, and heart (5–8). The ClC-3 chloride channels are also present in undifferentiated human pre-adipocytes, which can be inhibited by the chloride ion blocker tamoxifen (9). Experimental evidence from animal models also suggested that mice after *Clcn3* knockout exhibited changes in systemic metabolism, such as leptin and insulin secretion (6, 10–13), and can inhibit atherosclerotic lesion development (14).

There is not yet sufficient research evidence and definitive understanding that *Clcn3* deficiency directly ameliorates obesity and its’ possible mechanism. Our present study demonstrates that *Clcn3* deficiency mice are associated with a remarkably beneficial metabolic phenotype because the knockout of *Clcn3* reduces weight gain induced by the high-fat diet, visceral fat accumulation, and glucose and lipid disorders. Notably, we first used transcriptome to explore the *Clcn3* targeted gene pathway, and it shows that metabolically beneficial genes significantly upregulate. The western blotting indicated that *Clcn3* deficiency may inhibit obesity through the AMPK-UCP1 axis, which could become a potential target for improving metabolic dysfunction and obesity.

## 2 Research design and methods

### 2.1 Generation of *Clcn3*^−/−^ mice

*Clcn3* knock-out mice were obtained from the mating of heterozygous mice, which were produced via the CRISP-cas9 technique (Cyagen, China).

*Clcn3*^+/−^ genotyped female mice and *Clcn3*^+/−^ genotyped male mice were used to breed. Each male and 3 female mice (7–8 weeks) were placed in the same cage for a long period. Female gestation results were observed and recorded every week. After birth, the wild-type (*Clcn3*^+/+^), heterozygous (*Clcn3*^+/−^), and homozygous mice (*Clcn3*^−/−^) were identified by a polymerase chain reaction when 4 weeks.

### 2.2 Animal studies

All animal experimental procedures were approved by the Laboratory Animal Administration Committee of Xi’an Jiaotong University (2021-1499) and in compliance with the ARRIVE guidelines. Four weeks after the birth of mice, 1 mm of mouse tail was cut into a 1.5 mL centrifuge tube, 97 μL of lysis solution (1.37 g KCl, 1.2 g Tris, 1 mL TritonX-100, dissolved in 1 L of purified water, and concentrated hydrochloric acid was added to adjust the pH to 9.0) and 3 μL of 20 mg/mL of proteinase K were added, and lysis was performed at 56°C overnight. On the second day, heated at 98°C in a metal bath for 15 min, proteinase K was denatured, and lysis was terminated. 10,000 r centrifugation was performed for 15 min, and the supernatant was removed to obtain mouse genomic DNA.

A total of 3 primers are required for PCR identification of mouse genotypes, the length of the PCR product for the WT *Clcn3* was 769 bp, for which the sequences of primers were TTAGTGCTGGCTGTGGCATC (F1) and TCCCAGAGACAATGAGGCTAAGG (R). The PCR product of 654 bp was for the mutated *Clcn3* and the sequences of primers were TCTGA TGGGGACTAAGTATGCAG (F2) and TCCCAGAGACAATGAGGCTAAGG(R).

Because most of the *Clcn3*^−/−^ mice were female, we all used female mice in the following experiments. The female mice (4 weeks old, total of 24 mice) were divided into 4 groups and fed the high-fat diet (HFD, 40% calories from fat, D12109C Research Diets Inc., United States) or a normal chow diet (NC, 10% calories from fat, D12450; Research Diets Inc., United States) for 16 weeks, living in the SPF grade animal room of the Animal Experiment Center of Xi’an Jiaotong University [SYXK 2018-001]. Breeding environment: mice were housed in separate cages with a maximum of 5 mice/cage, except during the metabolic measurements, under a 12 h dark/light cycle with *ad libitum* self-feeding in specific pathogen-free conditions of 55% humidity and 22°C.

For the intraperitoneal glucose tolerance test (PGTT), mice fasted for 12 h were injected with glucose (2 g/kg body weight, CAS-50997, Sigma-Aldrich, United States), the glucose of tail blood at 0, 15, 30, 60, 90, and 120 min after injection was measured using a glucometer (Accu-Check-Guide, Roche, Germany). For the insulin tolerance test (ITT), mice were fasted for 4 h with free access to water and injected with insulin (0.75 units/kg body weight, NovoRapid. Novo Nordisk, Copenhagen, Denmark). Blood was collected from the tail at 0, 15, 30, 60, 90, and 120 min, and glucose was measured using a glucometer Accu-Check-Guide, Roche, Germany. Then the time-concentration curves of blood glucose for different groups of mice were plotted and the area under the curve (AUC) was made and calculated.

### 2.3 Serum and tissue sample collection

The mice at the age of 20 weeks were anesthetized with 0.3% pentobarbital sodium solution and then euthanized by cervical dislocation; their liver, inguinal white adipose tissue (iWAT), epididymal WAT (eWAT), and brown adipose tissue (BAT) were dissected, photographed, and stored at −80°C; the blood obtained by eyeball extirpating was placed at 4°C for 2 h and then centrifuged at 4,000 rpm for 15 min to harvest serum. The principal parenchymal organs of mice were then dissected, weighed, photographed, and cryopreserved.

### 2.4 Blood measurement

The total cholesterol (TC), triglyceride (TG), high-density lipoprotein (HDL), and low-density lipoprotein (LDL) were examined by biochemical methods. The serums of the mice were assayed with an automated biochemical analyzer (BIOBASE, BK-280, China), using the TC kit (BIOBASE, 10412001H), HDL-C kit (BIOBASE, 10412002H), LDL-C kit (BIOBASE, 10412003H) and TG kit (BIOBASE, 10408002H).

### 2.5 Transcriptomics

The transcriptome of a 20-week mouse’s eWAT was sequenced by the Huayin Health Medical Group Company (Guangzhou, China). In brief, total RNAs were extracted using the TRIzol reagent (cat.265709, Life, United States) following the manufacturer’s instructions. After being qualified and quantified by the Agilent 2100 Bioanalyzer (Agilent, United States) and Nanophotometer^®^ (Implen, Germany), 1 μg RNA was used to purify mRNA via the VAHTS^®^ mRNA Capture Beads with Oligo dT (cat. N401-01, Vazyme Biotech, China). Subsequently, mRNA was reversely transcripted into the double-strand cDNA with VAHTS^®^ Universal V6 RNA-seq Library Prep Kit (cat.NR604, Vazyme Biotech, China). Then, cDNA was added to an A base and digested with the UDG enzyme. Favorable cDNA fragments were selected for PCR amplification to establish the cDNA library. Finally, 2 × 150 bp paired-end sequencing was performed on a NovaSeq^™^ 6000 system (Illumina Corporation, United States) under the vendor’s guidance.

Raw reads proceeded to quality control, and then, clean data were obtained. After being blasted with the mouse reference genome (mmu10), the algorithm of RNA-Seq by Expectation Maximization (RSEM) was adopted to compute the gene expressions, of which the differential expression analysis between *Clcn3*^+/+^ and *Clcn3*^−/−^ mice was done with edgeR (Robinson, United States). The genes with |log2Ratio| ≥1 and *p*-value <0.05 were regarded as differentially expressed genes (DEGs), which were further explored by the enrichment of Kyoto Encyclopedia of Genes and Genomes (KEGG) pathways. The RNA sequencing data has been deposited to the SRA database, the accession number is PRJNA1087716.

(The SRA records link: https://www.ncbi.nlm.nih.gov/sra/PRJNA1087716).

### 2.6 Western blotting

Total proteins were extracted with RIPA buffer (P0013, Beyotime, China) and quantitated by the BCA assay (#20201ES76, Yeasen, China). Following boiled with the loading buffer, the denatured proteins were equally loaded and separated in SDS-PAGE gels. Subsequently, they were transferred onto PVDF membranes (ISEQ00010, Millipore, United States). The membranes were blocked with 5% BSA for 1 h and incubated with the diluted solutions of the primary antibodies at 4°C overnight (1:1,000 for UCP1, #14670S, CST, USA; 1:2000 for AMPK and p-AMPK Duet, #8208S, CST, USA; 1:5000 for β-actin, #20536-1-AP, Proteintech, China). Following washing of the non-specifically bound primary antibodies, the membranes were immersed in solutions with the corresponding secondary antibody for 1 h at room temperature (1,5,000 for anti-rabbit IgG, #RS0002, Immunoway, United States). The protein bands were finally detected by the enhanced chemiluminescent HRP substrate kit (WBKLS0100, Millipore, United States). The total intensity of each band was measured by ImageJ software (ImageJ, v1.8.0, United States), and was normalized according to the β-actin expression.

### 2.7 Statistical analysis

All data are expressed as the mean ± SEM. Unpaired student’s *t*-test was used to compare 2 groups. The normality and homogeneity of variance of the data were verified using the Shapiro–Wilk test and Levene’s test, respectively. If a normal distribution could not be assumed, the nonparametric Mann–Whitney *U*-test was performed. In cases of unequal variance, we applied Welch’s correction test. The statistical significance was evaluated via analysis of variance using GraphPad Prism8 software and SPSS Statistics 25 software. *p* < 0.05 was considered statistically significant.

## 3 Results

### 3.1 *Clcn3* deficiency reduced HFD-induced obesity

**Figure 1:**
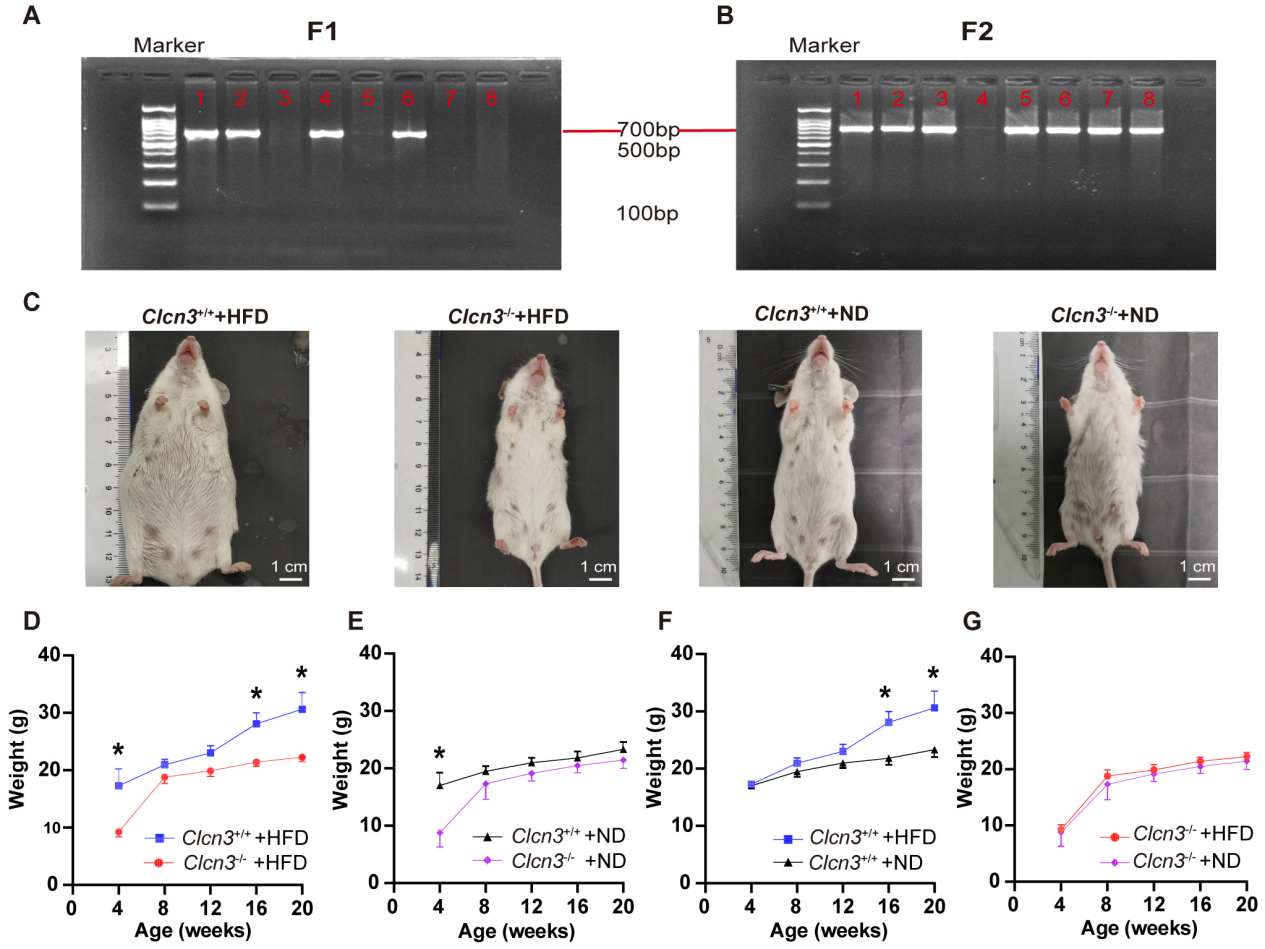
*Clcn3* deficiency ameliorates HFD-induced obesity. **(A)** Results of genotype identification of partial mice about F1. **(B)** Results of genotype identification of partial mice about F2. **(C)** Photos of mice from different groups. **(D)** Body weight changes with age in mice from different groups. ^*^p < 0.05.

As shown in Figures 1A,B, DNA was extracted from the tail of 4-week-old mice and amplified by PCR to determine the *Clcn3* genotype: there was only one band of 769 bp PCR product for *Clcn3*^+/+^ mice and one 654 bp band for *Clcn3*^−/−^ mice, and two bands indicated the heterozygous mice. Figure 1C showed the body picture in different groups of female mice of 20-week-old. After feeding with the high-fat diet, *Clcn3*^−/−^ mice were smaller compared to the *Clcn3*^+/+^ mice. However, after feeding with a normal diet, *Clcn3*^−/−^ mice and *Clcn3*^+/+^ mice had a similar body type.

In high-fat diet groups, compared with the *Clcn3*^+/+^ mice in 4 weeks, the weight of the *Clcn3*^−/−^ mice had already shown a significant decrease, and the *Clcn3*^−/−^ mice were significantly lighter than the *Clcn3*^+/+^ mice in 16 and 20 weeks (Figure 1D, *n* = 6, *p* < 0.05). In a normal diet, although there were already differences in body weight of mice at 4 weeks, after 16 weeks of feeding, the *Clcn3*^+/+^ mice and the *Clcn3*^−/−^ mice had no significant difference (Figure 1E, *n* = 6, *p* < 0.05). It is suggested that *Clcn3* deficiency reduced HFD-induced obesity, but had no significant effect in normal diet.

Compared with the two dietary conditions, there was no significant difference in body weight of mice in 4 weeks. After 12 weeks of feeding, the *Clcn3*^+/+^ mice in the high-fat diet were significantly weigher than *Clcn3*^+/+^ mice with a normal diet (Figure 1F, *n* = 6, *p* < 0.05). However, the weight of *Clcn3*^−/−^ mice with the high-fat diet had no significant increase compared with the normal diet (Figure 1G, *n* = 6, *p* > 0.05). The above results showed that the high-fat diet successfully induced obesity in *Clcn3*^+/+^ mice, but it did not cause obesity in *Clcn3*^−/−^ mice. Thus, *Clcn3* knockout could resist weight gain associated with the high-fat diet.

### 3.2 *Clcn3* deficiency reduced fat, especially visceral fat

**Figure 2:**
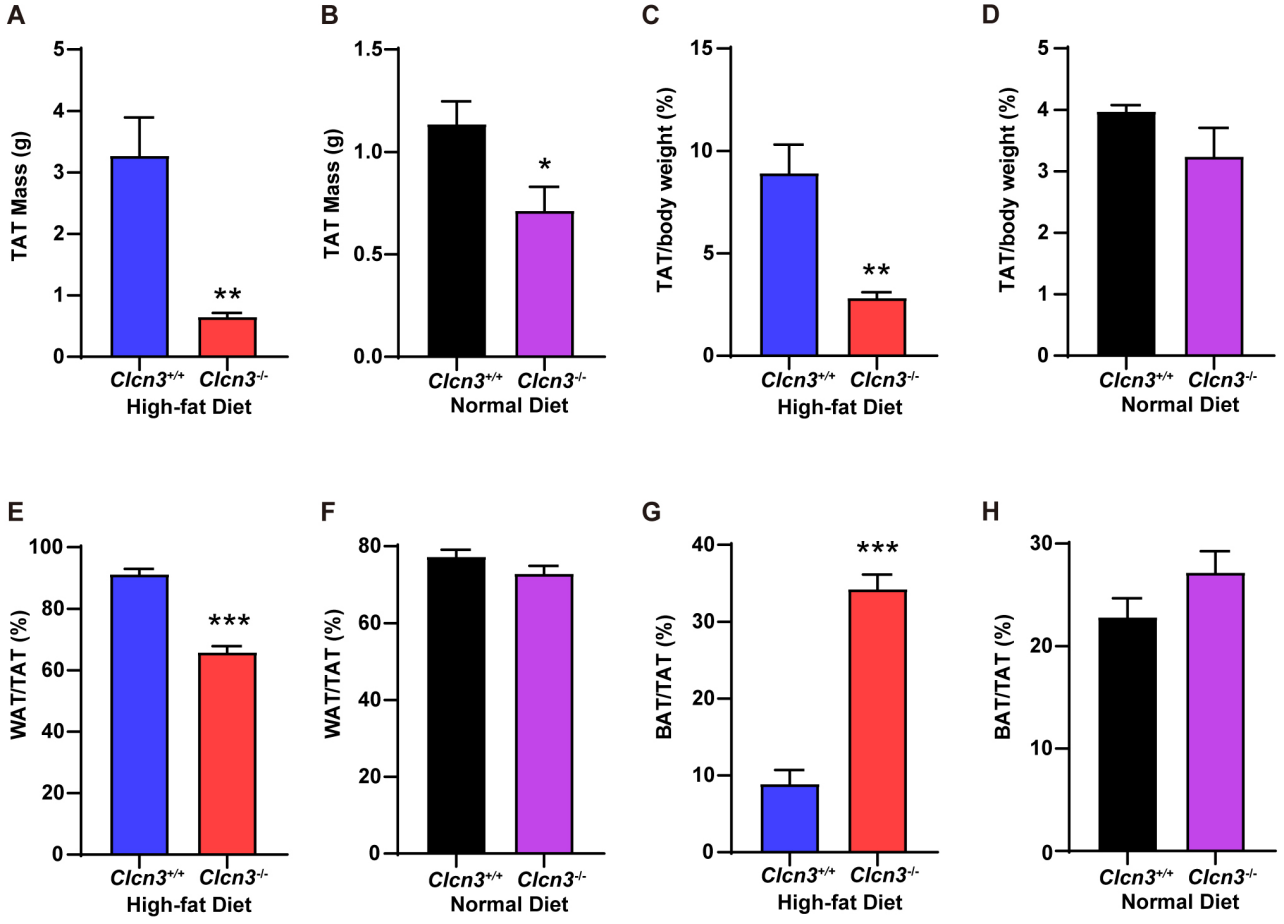
*Clcn3* deficiency ameliorates HFD-induced increase of white adipose tissue meanwhile increasing brown adipose tissue. **(A)** The weight of TAT from HFD groups. **(B)** The weight of TAT from ND groups. **(C)** The TAT percentage in body weight from HFD groups. **(D)** The TAT percentage in body weight from ND groups. **(E)** The WAT percentage in TAT from HFD groups. **(F)** The WAT percentage in TAT from ND groups. **(G)** The BAT percentage in TAT from HFD groups. **(H)** The BAT percentage in TAT from ND groups. ^*^p < 0.05, ^**^p < 0.01, and ^***^p < 0.001.

In high-fat diet groups, the total adipose tissue (TAT) of *Clcn3*^−/−^ mice showed less TAT weight gain compared with *Clcn3*^+/+^ mice (Figure 2A, *n* = 6, *p* < 0.05). In normal diet groups, the TAT weight of *Clcn3*^−/−^ mice was also less than *Clcn3*^+/+^ mice (Figure 2B, *n* = 6, *p* < 0.05). Then calculating the TAT percentage in body weight, the *Clcn3*^−/−^ mice with high-fat diet showed a significant decrease (Figure 2C, *n* = 6, *p* < 0.05). But in the normal diet, there was no significant difference between *Clcn3*^−/−^ and *Clcn3*^+/+^ mice (Figure 2D, *n* = 6, *p* > 0.05). After calculating the TAT percentage in body weight to eliminate the effect of weight differences, the discrepancies between *Clcn3*^+/+^ and *Clcn3*^−/−^ mice in the normal diet disappeared. These observations showed that *Clcn3*^−/−^ deficiency reduced fat.

Furthermore, in high-fat diet groups, white adipose tissue (WAT) percentage in TAT of *Clcn3*^−/−^ mice significant decrease compared with *Clcn3*^+/+^ mice (Figure 2E, *n* = 6, *p* < 0.05), but had no significant difference in normal diet groups (Figure 2F, *n* = 6, *p* > 0.05). In the meantime, in high-fat diet groups, the brown adipose tissue (BAT) percentage in TAT of *Clcn3*^−/−^ mice was remarkably increased compared with *Clcn3*^+/+^ mice (Figure 2G, *n* = 6, *p* < 0.05), but did not have the significant difference in mice with normal diet (Figure 2H, *n* = 6, *p* > 0.05). These results showed that *Clcn3* deficiency alleviated HFD-induced increase of TAT and WAT while leading to a reduction in visceral fat, but increased the BAT. Taken together, these findings indicated that *Clcn3* deficiency exhibited a more metabolically beneficial phenotype.

### 3.3 *Clcn3* deficiency ameliorated HFD-induced dyslipidemia

**Figure 3:**
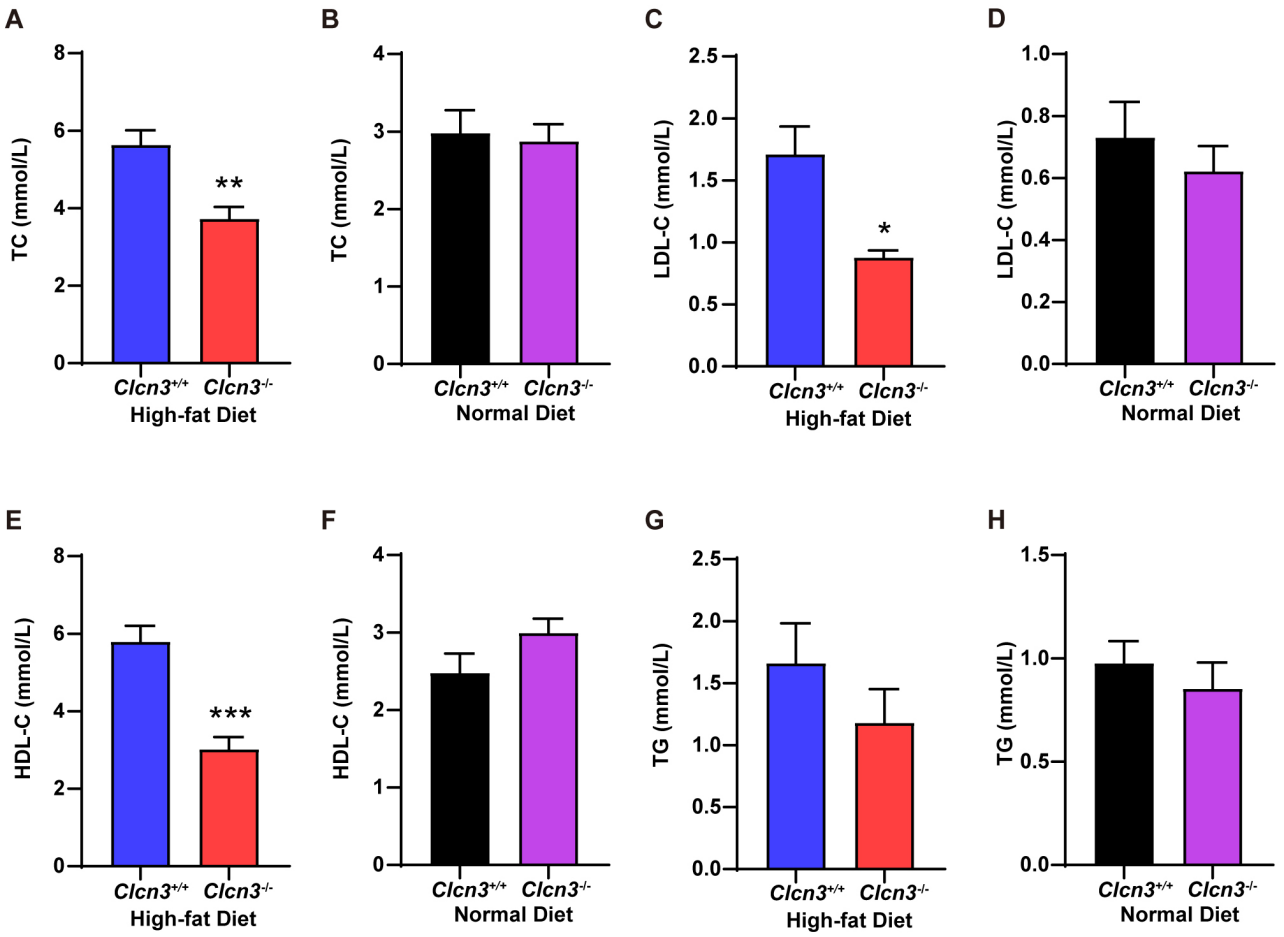
*Clcn3* deficiency ameliorates HFD-induced dyslipidemia. **(A)** The concentration of serum TC from HFD groups. **(B)** The concentration of serum TC from ND groups. **(C)** The concentration of serum LDL-C from HFD groups. **(D)** The concentration of serum LDL-C from ND groups. **(E)** The concentration of serum HDL-C from HFD groups. **(F)** The concentration of serum HDL-C from ND groups. **(G)** The concentration of serum TG from HFD groups. **(H)** The concentration of serum TG from ND groups. ^*^p < 0.05, ^**^p < 0.01, and ^***^p < 0.001.

Visceral obesity is associated with insulin resistance and dyslipidemia. We subsequently investigated whether *Clcn3* deficiency affects HFD-induced glucose and lipid metabolism disorders. After feeding high-fat diet, the concentration of serum TC from *Clcn3*^−/−^ mice was significantly less than *Clcn3*^+/+^ mice (Figure 3A, *n* = 6, *p* < 0.05), but normal diet groups had no significant difference (Figure 3B, *n* = 6, *p* > 0.05). In high-fat diet groups, compared with *Clcn3*^+/+^ mice, LDL-C is lower in *Clcn3*^−/−^ mice (Figure 3C, *n* = 6, *p* < 0.05). In the normal diet, LDL-C had no significant difference (Figure 3D, *n* = 6, *p* > 0.05). HDL-C was higher in *Clcn3*^+/+^ mice in the high-fat diet group (Figure 3E, *n* = 6, *p* < 0.05), but there was no significant difference in normal diet groups in different mice (Figure 3F, *n* = 6, *p* > 0.05). There were no significant differences in TG both in high-fat diet groups and normal diet groups (Figures 3G,H, *n* = 6, *p* > 0.05). These results support *Clcn3* deficiency ameliorated HFD-induced dyslipidemia.

### 3.4 *Clcn3* deficiency ameliorated HFD-induced glucose metabolism

**Figure 4:**
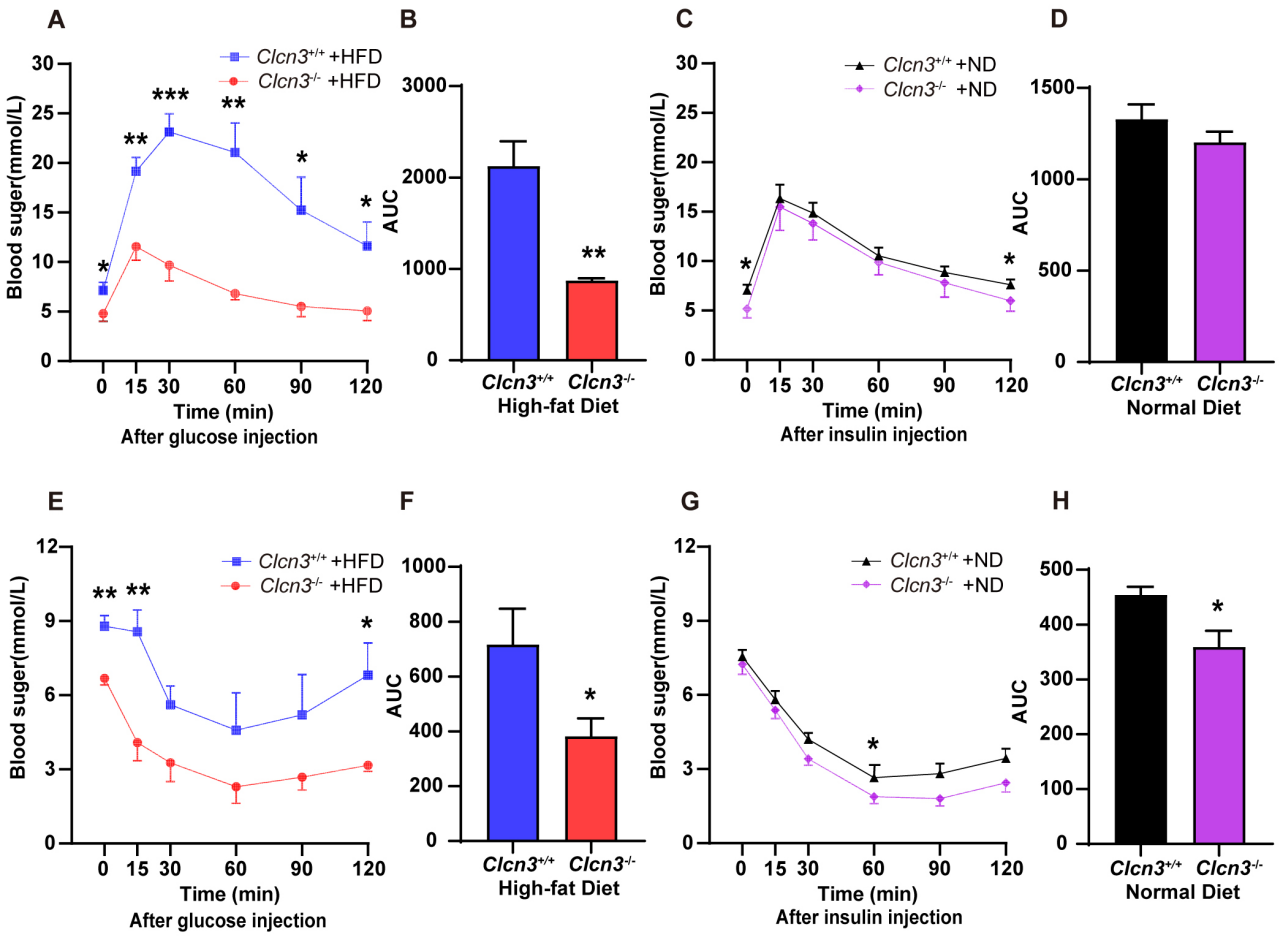
*Clcn3* deficiency ameliorates HFD-induced glucose metabolism disorders. **(A)** Glucose tolerance test (GTT) in *Clcn3*^+/+^ and *Clcn3*^−/−^ mice consuming with HFD (*n* = 5–6). **(B)** The area under the curve (AUC): analyses of GTT from HFD groups. **(C)** GTT in *Clcn3*^+/+^ and *Clcn3*^−/−^ mice consuming with ND (*n* = 5–6). **(D)** AUC: analyses of GTT from ND groups. **(E)** Insulin tolerance test (ITT) in *Clcn3*^+/+^ and *Clcn3*^−/−^ mice consuming with HFD (*n* = 5–6). **(F)** AUC: analyses of ITT from HFD groups. **(G)** ITT in *Clcn3*^+/+^ and *Clcn3*^−/−^ mice consuming with ND (*n* = 5–6). **(H)** AUC: analyses of ITT from HFD groups. ^*^p < 0.05, ^**^p < 0.01, and ^***^p < 0.001.

Next, we performed a glucose tolerance test (GTT), and compared *Clcn3*^−/−^ mice with the high-fat diet, the *Clcn3*^+/+^ mice with the high-fat diet showed a faster rise and lower drop of blood glucose. Conversely, the *Clcn3*^−/−^ mice with the high-fat diet had a lower rise and evenly drop of blood glucose. And every time point from the different groups’ mice had a significant difference (Figure 4A, *n* = 5–6, *p* < 0.05). Meanwhile, the area under the curve (AUC) of the *Clcn3*^−/−^ mice with the high-fat diet also showed a significant decrease compared with *Clcn3*^+/+^ mice with the high-fat diet (Figure 4B, *n* = 5–6, *p* < 0.05). The above description suggested that *Clcn3* deficiency could improve glucose metabolism disorders. In normal diet groups, it was only at 0 and 120 min that the two groups’ mice had the difference (Figure 4C, *n* = 5–6, *p* < 0.05), but the AUC had no significant difference (Figure 4D, *n* = 5–6, *p* > 0.05). These indicated that *Clcn3* deficiency can reduce the impaired glucose tolerance caused by high-fat diet, but had no significant effect on normal diet mice.

About the insulin tolerance test (ITT), after fasting for 4 h, *Clcn3*^+/+^ mice showed higher blood glucose at 0 min. Then the high-fat diet mice were injected with insulin, the blood glucose decreased in both *Clcn3*^+/+^ and *Clcn3*^−/−^ mice. The blood glucose levels were consistently lower in *Clcn3*^−/−^ mice compared with *Clcn3*^−/−^ mice, but there were significant differences only at 15 and 120 min (Figure 4E, *n* = 5–6, *p* < 0.05). The *Clcn3*^−/−^ mice with the high-fat diet had a smaller AUC compared with *Clcn3*^+/+^ mice (Figure 4F, *n* = 5–6, *p* < 0.05). In normal diet groups, the blood glucose’s remarkably difference showed only at 60 min, and AUC also had a significant difference (Figures 4G,H, *n* = 5–6, *p* < 0.05). These indicated that *Clcn3* deficiency can improve impairment of insulin sensitivity both in high-fat diet and normal diet.

### 3.5 The *Clcn3* deletion caused the transcriptional changes of metabolism-related genes

*Clcn3*^−/−^ mice had a lower body weight and visceral fat compared with *Clcn3*^+/+^ mice. Thus, adipose tissue for *Clcn3*^+/+^ and *Clcn3*^−/−^ mice were sequenced with RNA-seq to investigate the roles of *Clcn3* in the metabolism of mice in the high-fat diet.

**Figure 5:**
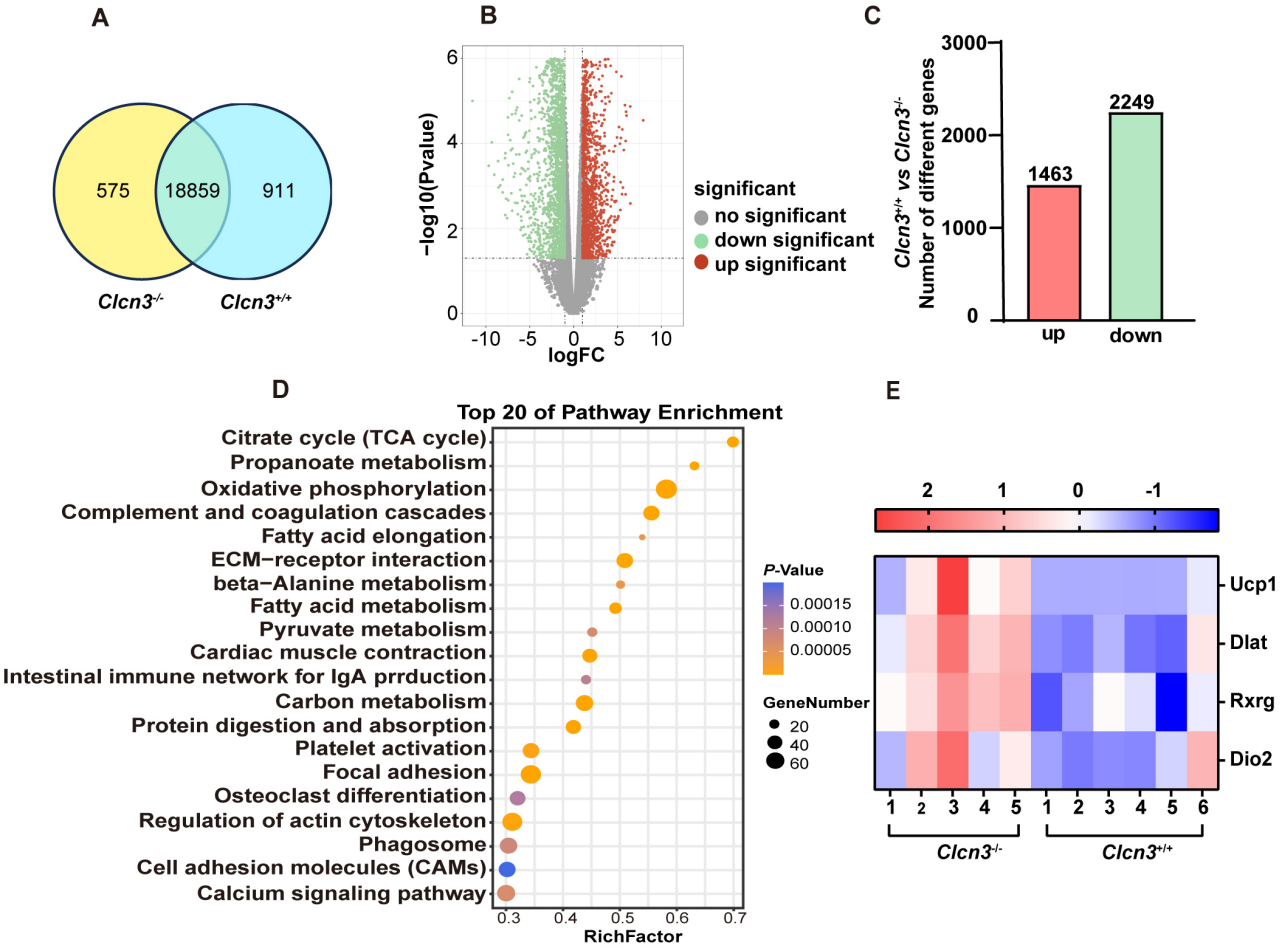
The *Clcn3* deletion caused the transcriptional changes of metabolism-related genes. Analysis of adipose sequencing gene in *Clcn3*^+/+^ and *Clcn3*^−/−^ mice with 20 weeks high-fat diet. **(A)** *Clcn3*^+/+^ and *Clcn3*^−/−^ mice common and unique genes Venn diagram. **(B)** Volcano plot of differentially expressed genes in *Clcn3*^+/+^ and *Clcn3*^−/−^ mice, red: differential genes with upregulated expression; green: down-regulated differential genes; gray: no significant difference. **(C)** *Clcn3*^+/+^ and *Clcn3*^−/−^ mice histogram of differential genes, red: differential genes with upregulated expression; green: down-regulated differential genes (*Clcn3*^−/−^ vs. *Clcn3*^+/+^ mice, *n* = 6, *p* < 0.05). **(D)** Top 20 KEGG enrichment pathways of DEGs. **(E)** Expression heatmap of DEGs (Ucp1, Dlat, Rxrg, and Dio2) related to metabolism between *Clcn3*^+/+^ and *Clcn3*^−/−^ mice.

Figure 5A Venn diagram showed the adipose tissue of *Clcn3*^+/+^ and *Clcn3*^−/−^ mice had 18,859 common genes. Compared with *Clcn3*^+/+^ mice, 3,712 DEGs in *Clcn3*^−/−^ mice were identified, including 1,463 upregulated genes and 2,249 downregulated genes (Figures 5B,C, *n* = 5–6, *p* < 0.05). All DEGs were analyzed by the enrichment of KEGG pathways. The top 20 of pathway enrichment were as shown in Figure 5D, the metabolism-related pathways, including oxidative phosphorylation, citrate cycle (TCA cycle), and fatty acid metabolism pathways (*p* < 0.05). Additionally, the PPAR signaling pathway was also significantly clustered. Then metabolism-related genes were found. Both *Dlat* and *Ucp1* were enriched in the citrate cycle and PPAR signaling pathways. Rxrg was enriched in PPAR signaling pathways. *Ucp1*, *Dio2*, *Dlat*, and *Rxrg* had significantly upregulated in *Clcn3*^−/−^ mice (Figure 5E, *p* < 0.05), which showed that *Clcn3*^−/−^ mice had a higher metabolic energy supply than *Clcn3*^+/+^ mice. Collectively, these results were consistent with lower body weight and less WAT phenotype in *Clcn3* knockout mice.

### 3.6 The effect of *Clcn3* deficiency on the activation of AMPK-UCP1 axis

**Figure 6:**
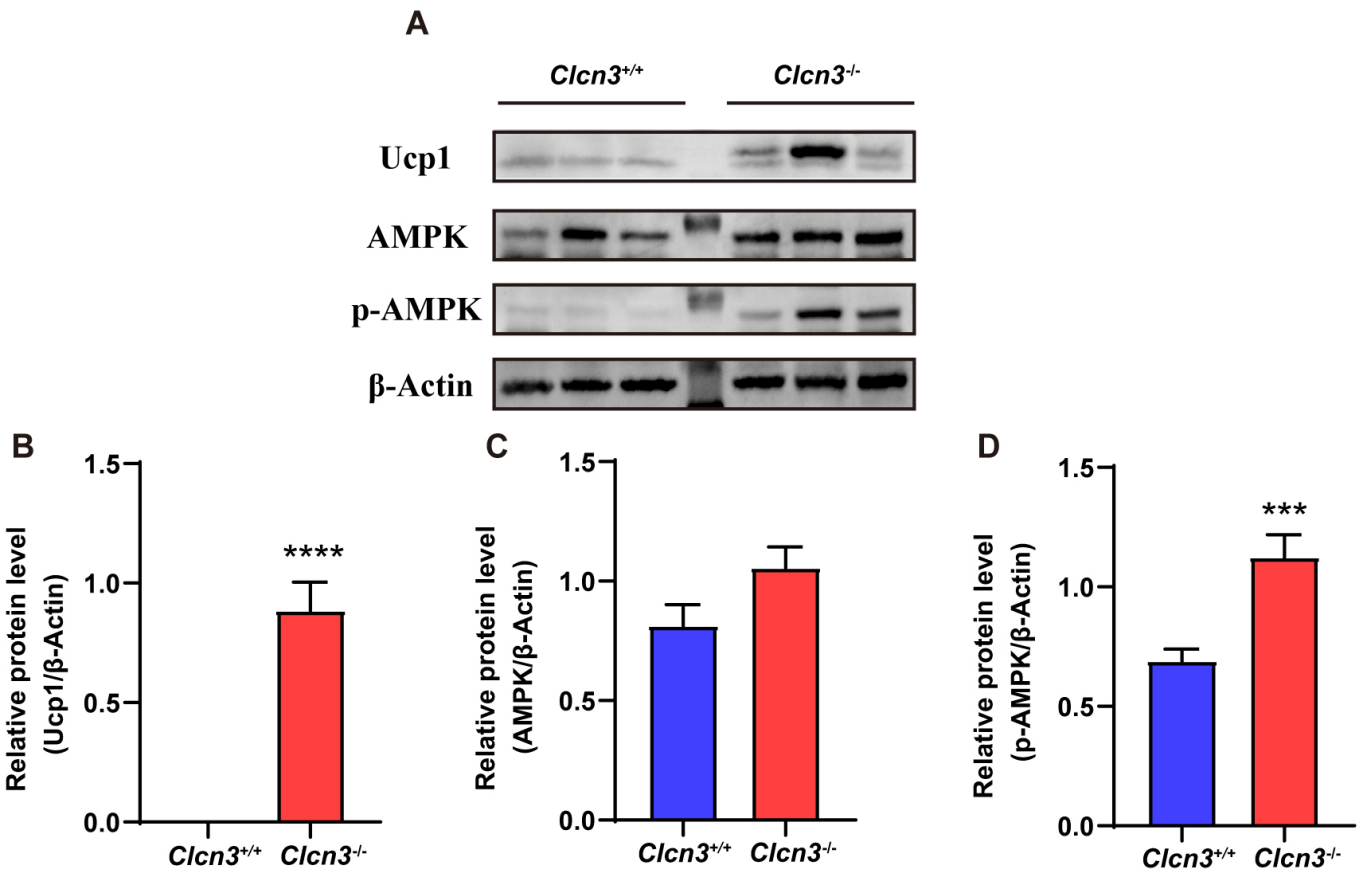
The *Clcn3* deficiency increased the expression of UCP1 and p-AMPK protein in mice. **(A)** Western blotting results of iWAT in high-fat diet *Clcn3*^+/+^ and *Clcn3*^−/−^ mice. **(B)** Relative protein level of UCP1/β-Actin. **(C)** Relative protein level of AMPK/β-Actin. **(D)** Relative protein level of p-AMPK/β-Actin. ^*^p < 0.05, ^**^p < 0.01, and ^***^p < 0.001.

The activation of the Adenosine monophosphate-activated protein kinase (AMPK) signal has been known to play a vital role in the regulation of thermogenesis in adipose tissue, especially the AMPK-UCP1 signaling axis. To identify the possible signaling pathway underlying the effects of *Clcn3* deficiency, the protein expression levels of UCP1, AMPK, and p-AMPK in iWAT were examined in high-fat diet mice. Compared with *Clcn3*^+/+^ mice, UCP1 and p-AMPK proteins were both significantly elevated in *Clcn3*^−/−^ mice (Figures 6A,B,D), although AMPK protein expression levels were comparable (Figures 6A,C). These data demonstrate that *Clcn3* deficiency might thereby promote adipose tissue thermogenesis via the pAMPK-UCP1 signaling axis to suppress diet induced obesity.

## 4 Discussion

High BMI, high cholesterol, high blood sugar, and high blood pressure are common metabolic disorder manifestations and exacerbate metabolic interactions. Several factors interact and increase the prevalence of metabolic diseases.

Animal experiments revealed that when fed by a normal diet 3-week-old *Clcn3*^−/−^ mice exhibited significantly reduced volumes for liver, kidney, heart, lung, and spleen compared with wild-type mice (15). In our study, the body weight of *Clcn3*^−/−^ mice was also significantly lower than that of *Clcn3*^+/+^ mice at the age of 4 weeks, but a lack of significant differences between the two groups at 8, 12, 16, and 20 weeks old when fed a normal diet. This result (Figure 1) indicated that the effect of *Clcn3* on the body weight of mice with a normal diet was mainly at a young age, our previous article also showed a similar result (16). *Clcn3* knockout delays the growth and development of mice. Obese patients have an increased risk of death, T2DM, and cardiovascular disease (17). They often have unhealthy diets. Thus, we give the high-fat diet as an experimental model to simulate the diet of obese people. The results showed that the body weight of *Clcn3*^−/−^ mice with the high-fat diet was lower than that of the *Clcn3*^+/+^ mice with the high-fat diet group at 16 and 20 weeks of age. Similar conclusions were also confirmed in a study of adipose tissue macrophage inflammation in mice (4). Thus, the results suggest that the *Clcn3* knockout can reduce weight gain from the high-fat diet. Interestingly, in the generation of *Clcn3*^−/−^ mice, we found most of the *Clcn3*^−/−^ mice were female. Stobrawa et al. found that disruption of *Clcn3* in mice entails severe neurodegeneration that, after a few months, leads to a conspicuous absence of the hippocampus (7). The hippocampus is the superior regulatory center of the hypothalamic-pituitary-ovarian axis, and hippocampal disruption leads to alterations in the gonadal axis, which may account for the manifestation of mostly female sex in *Clcn3* deficient mice. However, the alterations regarding the gonads of *Clcn3*^−/−^ mice deserve further exploration and study.

Adipose tissue plays an essential role in maintaining lipid, and glucose homeostasis, energy balance, and development of obesity (18). Dysfunctional adipose tissue could promote an inflammatory, hyperlipidemic, and insulin-resistant environment. Adipose tissue metabolic disorders further contribute to the development of T2DM and cardiovascular disease (19). Thus, we investigated whether *Clcn3* exerts its effects on adipose tissue to mitigate weight gain. White adipose tissue (WAT) primarily stores triglycerides and serves as the main reservoir for excess energy. Brown adipose tissue (BAT), known as a beneficial fat, exhibits non-shivering thermogenesis to burn energy, thereby reducing obesity (20). Brown and white adipose tissues work together to orchestrate energy balance and thermal regulation in endothermic animals. The accumulation of ‘brown-like’ adipocytes in WAT is referred to as “browning” or “beiging,” the activation of which upregulates *Ucp1* and other genes involved in energy expenditure in WAT. Browning of WAT is an adaptive and reversible response to environmental stimuli, including cold exposure, pharmacological agents such as β3-adrenergic receptor agonists and thiazolidinediones (TZDs), as well as various peptides and hormones (21). The findings shown in Figure 2 demonstrate that *Clcn3* deficiency mitigates the high-fat diet-induced increase in TAT and WAT percentage within TAT. Additionally, *Clcn3* deficiency leads to an increased BAT percentage within TAT. Consequently, the knockout of *Clcn3* may facilitate WAT browning to ameliorate obesity-related effects and subsequent metabolic dysfunction.

Adipocyte hypertrophy and excessive accumulation of adipose tissue can contribute to pathogenic effects on obesity, resulting in abnormal levels of circulating lipids. Dyslipidemia is a main cause of various metabolic diseases such as atherosclerotic cardiovascular disease, T2DM, and nonalcoholic fatty liver disease (22). HDL has been shown to protect against LDL oxidation, thereby preventing the generation of proinflammatory oxidized lipids (23). Notably, *Clcn3* deficiency prevents atherosclerotic lesion development in ApoE^−/−^ mice (24). The results depicted in Figure 3 proved that the knockout of *Clcn3* caused a decrease in LDL levels among mice fed with HFD. Interestingly, *Clcn3* deletion seemingly appears no significant influence on the TG level. In high-fat diet groups, HDL levels in *Clcn3*^−/−^ mice were lower than in *Clcn3*^+/+^ mice, which was considered to be due to an overall increase in TC levels in the *Clcn3*^+/+^ group. These studies collectively suggested that the high-fat diet induces expansion of adipose tissue, but *Clcn3* deficiency reduced this process and ameliorated lipid metabolism disorder. *Clcn3* will be a target in the treatment of metabolic diseases.

Obesogenic long-term metabolic disorders can lead to dysfunction of pancreatic βcell through various mechanisms. Glycolipid metabolism is the cornerstone of energy metabolism. *Clcn3* has been demonstrated to actively regulate glycolipid metabolism. The liver plays a crucial role in regulating glycolipid metabolism. The high-fat diet can induce fat accumulation and activate macrophage-driven inflammatory responses in the liver, resulting in the occurrence of obesity-related hepatic steatosis (25, 26). *Clcn3*, localized on insulin granules, could mediate insulin processing and secretion (10). The GTT (Figure 4) showed that *Clcn3* deletion delayed the response to increasing blood glucose levels and enhanced the efficiency of lowering blood glucose once initiated. Meanwhile, the ITT demonstrated that *Clcn3* deletion resulted in more moderate changes in blood glucose levels in mice. Collectively, these findings indicated that *Clcn3* deficiency improved insulin sensitivity, and glycolipid metabolism in diet-induced obese mice, which may be due to the lack of *Clcn3* ameliorating the systemic inflammatory response caused by the high-fat diet. *Clcn3* may be an early contributor to the development of obesity.

The eWAT transcriptional results (Figure 5) showed that the *Clcn3* deletion caused the 3,712 changes in genes, of which a part was enriched in the metabolism-related pathways, including oxidative phosphorylation, citrate cycle (TCA cycle), fatty acid metabolism, and PPAR signaling pathway. Studies have reported that cold exposure upregulated *Dlat*, and obese individuals have significantly lower expression of DLAT in adipose tissue. The phytochemical hyperforin triggers thermogenesis in adipose tissue via a Dlat-AMPK-UCP1 signaling axis to curb obesity (27). Cold through activation cAMP-PKA signaling pathway recruited PGC-1α to facilitate RXR heterodimerization to enhance *Ucp1* gene transcription. Then, UCP1 dispersed the released fatty acids to generate heat, and *Dio2* is also considered to be one of the small molecules for fat combustion (28). The energy metabolism-related genes *Ucp1*, *Dio2*, *Dlat*, and *Rxrg* were then screened by us, which all had significantly upregulated in *Clcn3*^−/−^ mice with the high-fat diet.

UCP1 is a mitochondrial protein specific to brown adipose tissue (BAT), and it uncouples cellular respiration and mitochondrial ATP synthesis to dissipate energy in the form of heat. UCP1 could mediate the functions of brown and beige fat, which promote anti-obesity and anti-diabetic effects when activated (29–31). Increase of UCP1 in the WAT of *Clcn3* knockout mice, suggesting that UCP1 may be a target for *Clcn3* deficiency to prevent obesity. According to previous reports, AMPK upregulates and activates PGC1a, which binds to the promoters of UCP1 and other thermogenic genes to co-activate their transcription (32, 33). Given the well-known role of AMPK as a sensor of intracellular energy state by regulating fatty acid metabolism and thermogenesis in adipose tissue (34), we investigated whether *Clcn3* deficiency was able to activate AMPK. Our results of western blotting (Figure 6) showed that UCP1 and p-AMPK protein had significantly elevated expression in *Clcn3*^−/−^ mice. Thus, we consider that UCP1 may be regulated by AMPK, and AMPK phosphorylation promotes increased UCP1 expression, which promotes adipose thermogenesis, and is responsible for the lighter body weight and less adiposity exhibited by *Clcn3* knockout mice.

In conclusion, *Clcn3* deficiency ameliorates HFD-induced obesity, meanwhile, improves glucolipid metabolism disorders, and the impairment of insulin sensitivity, which may be because of the AMPK-Ucp1 axis. These results suggest that modulation of *Clcn3* may provide an appealing therapeutic target for obesity and associated metabolic syndrome. Meanwhile, metabolic risk factors can be prevented and treated by exercising and improving their dietetic habit.

## Data availability statement

The datasets presented in this study can be found in online repositories. The RNA sequencing data has been deposited to the SRA database; the accession number is PRJNA1087716.

## Ethics statement

The animal study was approved by the Laboratory Animal Administration Committee of Xi’an Jiaotong University (2021-1499). The study was conducted in accordance with the local legislation and institutional requirements.

## Author contributions

SD: Data curation, Formal analysis, Investigation, Methodology, Validation, Visualization, Writing – original draft, Writing – review & editing. BL: Data curation, Formal analysis, Investigation, Methodology, Validation, Visualization, Writing – original draft, Writing – review & editing. SC: Validation, Visualization, Writing – review & editing. YC: Validation, Writing – review & editing, Visualization. YH: Conceptualization, Methodology, Resources, Validation, Visualization, Writing – review & editing. LF: Conceptualization, Data curation, Formal analysis, Funding acquisition, Investigation, Methodology, Project administration, Resources, Software, Supervision, Validation, Visualization, Writing – review & editing.
